# Survey of smokers' reasons for not switching to safer sources of nicotine and their willingness to do so in the future

**DOI:** 10.1186/1477-7517-6-14

**Published:** 2009-07-02

**Authors:** Karyn K Heavner, Zale Rosenberg, Carl V Phillips

**Affiliations:** 1 School of Public Health, University of Alberta, 8215 112 St Suite 215, Edmonton, AB, T6G 2L9, Canada

## Abstract

Despite the well-known risks of smoking, policy, social pressure, and accessible cessation programs, tens of millions of North American adults continue smoking rather than quitting or switching to less harmful non-combustion nicotine products. We surveyed people smoking in public in Edmonton, Canada (n = 242, year = 2007) to investigate smokers' reasons for resisting switching to low-risk nicotine sources. 43% had used low-risk products (mostly pharmaceutical nicotine). 75% indicated willingness to consider switching to low-risk products. Smokers cited similar reasons for not switching to smokeless tobacco and pharmaceutical nicotine, largely based on misinformation. Accurate risk information may lead many to try low-risk nicotine products.

## Background

Despite massive education campaigns and legal restrictions, the prevalence of habitual nicotine use among adults in Canada and the United States (US) remains at about one quarter of the population, and most use the deadliest source of nicotine, cigarettes [1-3]. Smokeless tobacco (ST) causes roughly 1/100^th ^the health risks of smoking (see our website for more details: ) [4] and long term use of pharmaceutical nicotine is believed to be similarly low risk though we lack direct evidence, so can only extrapolate assuming the risks are similar to those from ST. Thus, persuading smokers who cannot or will not quit using nicotine to switch to alternative sources is almost as beneficial to their health as getting them to quit entirely. But unlike in Sweden, where ST has largely replaced smoking [5,6], there has been a very limited shift from smoked to smokeless sources of nicotine in North America. Even heavily-promoted pharmaceutical products have generated limited interest. For example, only 25% of participants in a national sample of adult smokers in the United States had ever used nicotine patches and 22% had ever tried nicotine gum, despite the fact that about 95% of participants were aware of these products [7].

One of the barriers to smoking cessation via product switching is misinformation about ST and pharmaceutical nicotine products. Most (67%) people in a telephone survey in the US [8] and 59.8% of a sample of nurses [9] mistakenly believed that nicotine is the main cause of tobacco-related cancers. Surveys of smokers and college students in North America found that fewer than 15% realize that ST is less harmful than smoking [10-12]. In addition, a study found that most (75%) male US military recruits believe that switching from smoking to ST does not reduce tobacco users' risk [13]. Many smokers have similar misconceptions about the health risks from using pharmaceutical nicotine products [7,8]. The present study was designed to specifically investigate smokers' amenability to harm-reducing product switching, including consideration of switching to ST and pharmaceutical nicotine and potential willingness to switch in the future, as well as to partially assess the informational barriers to switching.

## Methods

The research team enrolled a convenience sample of adult smokers in public outdoor areas in Edmonton, Alberta where people were smoking during five days in September 2007. The public outdoor areas included designated smoking areas outside of office buildings, shopping malls, bars, restaurants and construction sites. The researchers systematically approached the first person they saw smoking or starting to smoke upon completing the previous survey. While the exact population we sampled from is difficult to define, this was intended to provide a reasonable cross-section of the target population for our harm reduction education efforts in Edmonton; adult smokers who are literate in English and engaged in civic life.

Our plan had been for the survey to precede the launch of a harm-reduction-based marketing effort for a new ST product in Edmonton. The survey was conducted shortly after the product rollout due to delays by the university health research ethics board which approved the study. However, limited marketing preceded the survey and there was limited awareness of the new product, so we do not believe that the product launch had a major impact on subjects' perceptions.

Upon confirming that potential participants were at least 18 years old, they were given an information sheet explaining the survey and their rights as a research participant. People who were eligible and agreed to participate completed a one-page, anonymous, self-administered survey. The researchers read the survey to people who wished to complete the survey but were visually impaired or indicated that they could not complete a self-administered survey. The research team defined terms on the survey, such as snus and medicinal nicotine, if the participants indicated that they did not understand them. Four hundred thirteen adult smokers were approached, 242 of whom completed the survey (response rate: 59%).

The survey assessed participants' use of cigarettes, ST and pharmaceutical nicotine; reasons for not switching to ST or pharmaceutical nicotine for harm reduction; and interest in using hypothetical reduced harm nicotine products. Some questions were based on the common beliefs and misperceptions about tobacco and nicotine that are reported in the literature or that we have observed in our experience as educators. To assess the stated willingness to switch to a reduced harm product with minimal misinformation baggage, we asked participants if they would consider switching from smoking to two hypothetical products; those products were described as providing nicotine in a way that was almost as satisfying as smoking and could be used without anyone noticing, with one reducing the health risks by 99% and the other by 50%. The subjects were never told that the "hypothetical" 99%-reduction product described existing ST products, nor was any other potentially persuasive information offered. Extensive epidemiological evidence shows that this is a good approximation of the comparative risk for ST, and many smokers have found the products to be a satisfying substitute source of nicotine. Though there are no useful epidemiological studies about long-term use of pharmaceutical nicotine, there is no reason to believe that these products pose greater risk than ST, so this can be used as a best guess about their effects also. Approved usage of existing pharmaceutical products is usually judged to be a poor substitute in terms of nicotine delivery for tobacco harm reduction. However, electronic cigarettes, which were not yet well-known at the time of the survey, are quite liked by many smokers. The survey and data are available at 

SAS (version 9.1, SAS Institute, Cary, North Carolina) was used for data cleaning and analysis. Our analysis methodology called for simply reporting univariate summaries of responses as well as the bivariate analysis that appears below. Male and female participants were compared. We also compared reasons why participants did not previously consider switching to safer nicotine products and their willingness to consider the hypothetical products. Tests of statistical significance were not presented because these are only appropriate when the purpose of a study is to test a hypothesis rather than estimate prevalence. We present 95% confidence intervals (CIs) to give readers an indication of the robustness of the results but remind readers that interpretation of these is still only indicative of random error. In surveys such as this, and indeed most epidemiology, the largest sources of error when extrapolating the results to some population of interest are likely non-random, such as systematic differences between the study sample and target population and measurement error, including misinterpretation of survey questions and misclassification due to provision of answers that are perceived to be "correct" or socially desirable.

## Results and discussion

The results appear in Tables 1, 2 and 3. Participants' current smoking status was based on observing them smoking before, during, or soon after completing the survey. Their demographic characteristics and their history of using nicotine products are described in Table 1. Overall, 43% of participants had used low-risk nicotine products (mostly pharmaceutical nicotine products). Four people had attempted to use ST as a smoking cessation method. Most participants had previously attempted cessation or stated an expectation of quitting in the future. This survey of current smokers obviously could offer no assessment of successful cessation. Reported average consumption was 15 cigarettes per day. (The midpoint was used for participants who entered a range. Two responses given in packs-per-day were converted by multiplying by 20, though the number of cigarettes per pack in Canada varies.)

**Table 1:** Participants' Demographic Characteristics and Usage of Products Containing Nicotine

Age		
Mean (95% CI)	38	(36, 40)
Median (range)	39	(18–67)
	n	%

Male	106	44%
		
Approximate number of cigarettes currently smoked per day		
Mean (95% CI)	15	(14, 16)
Median (range)	15	(0–50)
		
Used smokeless tobacco at least 10 times		
Yes	25	11%
No	207	89%
		
Used pharmaceutical nicotine at least 10 times		
Yes	84	36%
No	152	64%
		
Ever tried to quit smoking		
Yes	217	90%
No	24	10%
Cessation methods that smokers previous tried^1^		
Stopped all at once ("cold turkey")	163	67%
Gradually decreased the number of cigarettes smoked	120	50%
Counselling or a stop-smoking clinic or program	14	6%
Switched to chewing tobacco or snuff	4	2%
Medicinal nicotine products	84	35%
Zyban/Wellbutrin/Buproprion or other medication	61	25%
Other methods	20	8%
		
Expect to quit smoking within the next 2 years		
Yes	162	71%
No	65	29%

^1^Limited to participants who ever tried to quit smoking.

**Table 2:** Barriers to Using Less Harmful Sources of Nicotine

	Smokeless tobacco (n = 236)	Pharmaceutical nicotine products (n = 236)	Smokeless tobacco or pharmaceutical nicotine products (n = 234)
	
	% Yes (95% CI)	% Yes (95% CI)	% Yes (95% CI)
*Never *considered quitting smoking, but continuing to use nicotine, by switching from smoking to this product?	90% (86%, 94%)	60% (54%, 66%)	56% (50%, 62%)

Reasons for not considering switching^1^	(n = 212)	(n = 142)	(n = 131)
I believe that using *tobacco *in any form is as bad for you as smoking.	49% (42%, 56%)	NA	NA
I believe that using *nicotine *in any form is as bad for you as smoking.	42% (35%, 49%)	43% (35%, 51%)	51% (43%, 60%)
I believe that using smokeless tobacco is socially unacceptable or gross (because you have to spit or it makes a mess in your mouth).	41% (34%, 47%)	NA	NA
There are things I enjoy about smoking besides just getting nicotine.	35% (28%, 41%)	33% (25%, 41%)	43% (34%, 51%)
I believe that smokeless tobacco would increase my risk of oral (mouth) cancer.	34% (28%, 40%)	NA	NA
I believe that these products are more likely to cause addiction than smoking.	14% (9%, 19%)	14% (8%, 20%)	22% (15%, 29%)
Smokeless tobacco is hard to use.	11% (7%, 16%)	NA	NA
Smoking is important to my social life.	10% (6%, 14%)	8% (4%, 13%)	12% (7%, 18%)
I tried these products but I did not find them satisfying.	3% (1%, 6%)	9% (4%, 14%)	11% (5%, 16%)
The labels on medicinal products say they should only be used for a limited period of time.	NA	6% (2%, 9%)	NA
Medicinal nicotine products are too expensive.	NA	5% (1%, 8%)	NA

^1^Limited to participants who never considered switching from smoking to this product.

NA – Not applicable.

**Table 3:** Variation in willingness to switch to "hypothetical" reduced risk nicotine products between those who did and did not cite particular reasons for not previously switching to safer products

		% would consider switching to a hypothetical reduced risk nicotine product in the future ^2^
Reasons for not *previously *considering switching to *ST or pharmaceutical nicotine *to stop smoking but continuing to use nicotine^1^		(n = 130)

I tried switching to ST/pharm. nicotine but did not find it satisfying.	Yes	79% (57%, 100%)
	No	59% (51%, 68%)
I believe ST/pharm. nicotine is likely to cause addiction than smoking.	Yes	69% (52%, 86%)
	No	59% (50%, 69%)
I believe that nicotine in any form is as bad as smoking.	Yes	67% (55%, 78%)
	No	56% (44%, 68%)
There are things I enjoy about smoking besides just getting nicotine.	Yes	60% (47%, 73%)
	No	63% (52%, 74%)
Smoking is important to my social life.	Yes	47% (21%, 72%)
	No	63% (55%, 72%)

Reasons for not *previously *considering switching to *ST *to stop smoking but continuing to use nicotine^1^		(n = 211)
I believe that using *tobacco *in any form is as bad for you as smoking.	Yes	76% (68%, 84%)
	No	70% (61%, 79%)
I believe that using smokeless tobacco is socially unacceptable or gross (because you have to spit or it makes a mess in your mouth).	Yes	79% (70%, 88%)
	No	69% (61%, 76%)
I believe that smokeless tobacco would increase my risk of oral (mouth) cancer.	Yes	81% (71%, 90%)
	No	69% (61%, 77%)
Smokeless tobacco is hard to use.	Yes	83% (67%, 98%)
	No	72% (65%, 78%)

^1^Limited to smokers who had not previously considered switching to ST/pharmaceutical nicotine and responded to the questions about whether they would consider switching to hypothetical reduced risk nicotine products in the future.

^2^Would consider switching to a nicotine product that is 50% or 99% less harmful than cigarettes.

Smokers' attitudes towards pharmaceutical nicotine products and ST are described in Table 2. Only 10% of those surveyed had considered switching to ST, and 40% had ever considered switching to pharmaceutical nicotine products. Those who had *never *considered switching to these products were asked to specify why not. The reasons given by those who had not considered switching to pharmaceutical nicotine products and ST were similar. The most common reasons given for not switching to either ST or pharmaceutical nicotine are popular fallacies. For example, many participants did not switch to safer products because they believed that tobacco or nicotine in any form is as harmful as smoking and that ST is more likely to cause oral cancer than smoking. This is consistent with a previous study in which university students who smoked attributed a mean of 16% of the risks of cigarettes to nicotine and 48% believed that ST definitely causes oral cancer (Geertsema K, Phillips CV, Heavner K. University Student Smokers' Perceptions of Risks and Barriers to Harm Reduction, Submitted, Available at: ).

**Figure 1:**
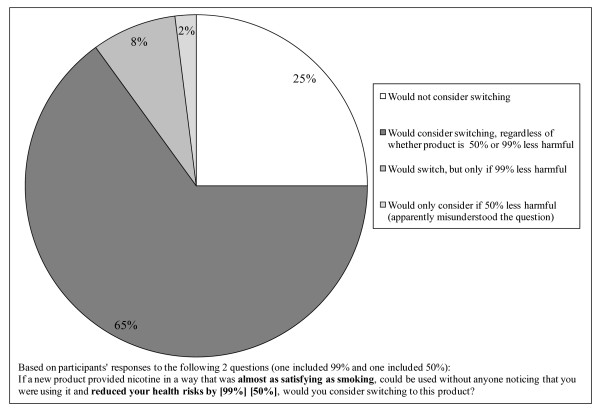
**Willingness to switch to reduced harm nicotine products (n = 238)**.

In response to the questions about the hypothetical reduced risk products, most participants (73%) were willing to consider switching to a product that is almost as satisfying as smoking but with 99% less risk than smoking (Figure 1). This is consistent with results from the aforementioned survey of student smokers, in which 64% would consider switching to a product with 1% of the health risks of smoking cigarettes (Geertsema K, Phillips CV, Heavner K. University Student Smokers' Perceptions of Risks and Barriers to Harm Reduction, Submitted, Available at: ). As discussed earlier, the hypothetical was intended to describe modern Western ST products. The description may overstate the satisfaction from most current pharmaceutical nicotine products, which are less satisfying substitutes for smoking than ST due to the higher price and slower delivery of nicotine if used as directed, but might apply to electronic cigarettes.

Few participants indicated that a 99% reduction in risk, but not a 50% reduction, would be worth considering. We did not attempt to tease out exactly how much reduction in risk was necessary to attract someone to consider switching, but merely wanted a very rough idea of whether respondents were very sensitive to the magnitude of the reduction. The answer was, with appropriate roughness, they were not very sensitive. Five people indicated that they were willing to switch to a product that reduced their risks by 50% but not to a 99% reduced risk product, possibly indicating a general innumeracy but possibly suggesting that the quantified reduced risk was misunderstood as the remaining risk, suggesting that care be taken when designing similar questions for future instruments. We did not attempt to further interpret the answers separately. Participants who were willing to consider switching to hypothetical products that were either 50% or 99% less harmful than smoking were classified as willing to consider switching in the remainder of the analysis.

There were several noteworthy differences between the male and female smokers in the sample. Female smokers were less likely to have used ST (5%, 95% CI: 1%, 8% versus 18%, 95% CI: 11%, 26%) and more likely to have used pharmaceutical nicotine at least 10 times (43%, 95% CI: 34%, 51% versus 26%, 95% CI: 18%, 35%), compared to males. Similarly, females were more likely to have considered switching to pharmaceutical nicotine than males (47%, 95% CI: 39%, 55% and 30%, 95% CI: 21%, 39%, respectively) and less likely to have considered switching to ST (8%, 95% CI: 3%, 12% and 14%, 95% CI: 7%, 20%, respectively). Many subjects who had not previously considered using ST had not consider switching because they believed it to be socially unacceptable or gross (44%, 95% CI: 35%, 53% of women and 36%, 95% CI: 26%, 46% of men). This reflects a lack of awareness about modern ST products, which can be used invisibly and without spitting. The difference between women and men probably reflects differing aversions to spitting rather than differing knowledge.

Males were more likely than females to have been deterred from switching to ST in the past because they believed that it is more addictive (19%, 95% CI: 11%, 28% versus 11%, 95% CI: 5%. 16%) and more likely to cause oral cancer (39%, 95% CI: 28%, 49% versus 31%, 95% CI: 23%, 39%) compared to smoking. Female smokers were more likely than males to consider switching to a reduced harm product; though this disparity was not great. Seventy-eight percent (95% CI: 71%, 85%) of females and 70% (95% CI: 61%, 79%) of males indicated that they would consider switching to a hypothetical reduced risk nicotine product. This result is interesting since in Sweden males have proven considerably more willing to switch [5,6]. Although no socioeconomic data was collected on the survey, the research team noted that a large proportion of the male smokers appeared to be construction and trades workers and a large proportion of the female smokers worked in downtown office buildings. The effect of these differences on the variations between the male and female smokers in this sample is unknown.

Participants' reasons for not previously switching to ST were associated with their willingness to consider switching to the hypothetical reduced risk nicotine products (Table 3). Those who had previously attempted to switch products were more likely to consider switching to the hypothetical product, as were those who had not tried to switch because of misperceptions about the health risks or addiction. Contrary to expectations, subjects' stating that there were factors other than nicotine that they liked about smoking seemed to have little effect on their willingness. There was a stronger association with stating that smoking was important to one's social life (though there were few who said yes to this). Seventy-nine percent of people who had not switched to ST because of concerns about social acceptability indicated that they would consider switching to hypothetical products that could be used discretely. Most (81%) of the smokers who did not switch to ST because they believe that it causes oral cancer were willing to consider switching to the hypothetical reduced risk product. This is consistent with the theory that smokers' reluctance to switch is attributable to erroneous beliefs about ST. In addition, 79% (95% CI: 73%, 85%) of smokers who expected to quit in the next two years would consider switching to the hypothetical reduced risk product, compared to 65% (53%, 77%) of smokers who did not expect to quit in the next two years.

## Conclusion

This study suggests that many adult smokers are interested in switching to safer forms of nicotine. While precise estimates from this small convenience sample would be inappropriate, the responses are consistent with reports of misperceptions about nicotine and tobacco in the literature, and strongly suggest that those misperceptions are major barriers to harm reduction. The failure to have already switched to existing low risk products, when contrasted with the willingness to switch to hypothetical products, seems largely explained by erroneous beliefs that tobacco and nicotine use are as inherently harmful as smoking. There was some evidence of dissatisfaction with existing alternatives, though much of the stated dissatisfaction about ST products further indicated erroneous beliefs about those products. It is true that nicotine products have non-nicotine aspects, such as flavor, physical comfort, ritual, and consumer self-identity, which make all substitutes for cigarettes imperfect, and it may be that consumers answering a hypothetical question do not recognize the impact of switching on these habits. On the other hand, the wide variety of low-risk products offer many options, and people often find it much easier to become used to a new ritual or habit than they think

This survey suggests that the barriers to switching are similar for ST and pharmaceutical nicotine, a somewhat ironic result. The widespread failure to understand that non-combustion nicotine sources pose a tiny fraction of the risk from smoking is largely attributable to the effective long-running disinformation campaign by anti-ST and anti-harm-reduction activists who are more concerned with promoting nicotine abstinence than public health [14,15]. These anti-tobacco lobbyists usually do not oppose pharmaceutical nicotine use, and indeed usually actively promote it. Ironically our results suggest that the anti-harm-reduction, "quit or die" messages have spilled over from ST to pharmaceutical nicotine, and have been equally effective in misleading smokers about the risks of both product types. This is an example of a general cautionary lesson: when opinion leaders manipulate people into doing something for reasons other than the real motives of the leaders (such as avoiding ST because of false beliefs about risk, rather than sharing their general despite of tobacco), the short-run results may be what the leaders intended, but the long run results will likely drift far from that.

Despite the disinformation, the survey suggests there is still great potential for tobacco harm reduction, making it probably the most significant untried public health intervention available in the West. Subjects who tried to switch before were not deterred from trying again and may be the most promising targets for encouragement to take this important step to improving their health. Multiple attempts and periodic relapses are common in the adoption of other safer behaviors such as safer sex, increasing physical activity and healthier eating. Anecdotal evidence from Sweden suggests that switching is very often successful as a gradual and non-monotonic process.

It appears that education about health risks and (particularly for women) the discreteness of modern ST products could lead a substantial portion of the smoking population to try switching. While the number who might actually make the effort to switch upon learning the truth would likely not be as high as those who were willing to effortlessly say "yes" to the hypothetical product, even a fairly small portion of that would represent millions of smokers. If efforts to actively convince smokers that there is no opportunity for harm reduction were to end, we would expect to see the change begin. If the resources that are currently devoted to misleading smokers about harm reduction were instead targeted at informing them that they have satisfying choices that are almost as good for their health as quitting entirely, a very large change could happen quite rapidly.

## Abbreviations

ST: Smokeless tobacco.

## Competing interests

The authors are interested in encouraging tobacco harm reduction (reducing the morbidity and mortality caused by tobacco use by encouraging smokers to switch to smokeless tobacco or other low-risk alternatives). As a result, they have an interest in doing research like this that explores factors that make tobacco harm reduction more or less likely to work. In addition to this actual substantial interest, the authors also have what some mistakenly consider to be the only real conflict of interest, funding from the private sector: Dr. Phillips and his research group (including Dr. Heavner and Mr. Rosenberg) are partially supported by an unrestricted (completely hands-off) grant to the University of Alberta from U.S. Smokeless Tobacco Company. The grantor is unaware of this study, and thus had no scientific input or other influence on it. Dr. Phillips has consulted for U.S. Smokeless Tobacco Company in the context of product liability litigation and subsequent to the completion of this paper became a member of British American Tobacco's External Scientific Panel advising on issues of tobacco harm reduction. Dr. Heavner owns a small amount of stock in Johnson & Johnson. Though these do and might (respectively) represent interests, and credibly influence *what *research we consider important, our interest in accurately assessing the barriers to harm reduction means it is not clear to us how these interests might be seen as justifying the knee-jerk accusation of bias – that we somehow altered the presentation of these results based on non-scientific interests – that we often face from the political activists who work to influence the science in this area.

## Authors' contributions

KH designed the survey. Data collection was conducted by KH, ZR and other students and researchers in CVP's research group. CVP supervised data collection and analysis. KH and ZR conducted the data analysis. All authors contributed to writing the manuscript and have read and approved the final manuscript.

## References

[B1] Hammond D (2005). Smoking behaviour among young adults: beyond youth prevention. Tob Control.

[B2] Lee DJ, Fleming LE, Arheart KL, LeBlanc WG, Caban AJ, Chung-Bridges K (2007). Smoking rate trends in U.S. occupational groups: the 1987 to 2004 National Health Interview Survey. J Occup Environ Med.

[B3] Tanuseputro P, Manuel DG, Leung M, Nguyen K, Johansen H (2003). Risk factors for cardiovascular disease in Canada. Can J Cardiol.

[B4] Phillips CV, Rabiu D, Rodu B (2006). Calculating the comparative mortality risk from smokeless tobacco versus smoking. American Journal of Epidemiology.

[B5] Rodu B, Stegmayr B, Nasic S, Asplund K (2002). Impact of smokeless tobacco use on smoking in northern Sweden. J Intern Med.

[B6] Stegmayr B, Eliasson M, Rodu B (2005). The decline of smoking in northern Sweden. Scand J Public Health.

[B7] Bansal MA, Cummings KM, Hyland A, Giovino GA (2004). Stop-smoking medications: who uses them, who misuses them, and who is misinformed about them?. Nicotine Tob Res.

[B8] Cummings KM, Hyland A, Giovino GA, Hastrup JL, Bauer JE, Bansal MA (2004). Are smokers adequately informed about the health risks of smoking and medicinal nicotine?. Nicotine Tob Res.

[B9] Borrelli B, Novak SP (2007). Nurses' knowledge about the risk of light cigarettes and other tobacco "harm reduction" strategies. Nicotine Tob Res.

[B10] O'Connor RJ, Hyland A, Giovino GA, Fong GT, Cummings KM (2005). Smoker awareness of and beliefs about supposedly less-harmful tobacco products. Am J Prev Med.

[B11] O'Connor RJ, McNeill A, Borland R, Hammond D, King B, Boudreau C (2007). Smokers' beliefs about the relative safety of other tobacco products: findings from the ITC collaboration. Nicotine Tob Res.

[B12] Smith SY, Curbow B, Stillman FA (2007). Harm perception of nicotine products in college freshmen. Nicotine Tob Res.

[B13] Haddock CK, Lando H, Klesges RC, Peterson AL, Scarinci IC (2004). Modified tobacco use and lifestyle change in risk-reducing beliefs about smoking. Am J Prev Med.

[B14] Phillips CV, Wang C, Guenzel B (2005). You might as well smoke; the misleading and harmful public message about smokeless tobacco. BMC Public Health.

[B15] Phillips CV, Bergen P, Guenzel B (2006). Persistent misleading health advice about smokeless tobacco on the Web. Poster presentation at the 11th World Congress on Internet in Medicine, Toronto, Canada.

